# Sociodemographic Correlates of Eye Care Provider Visits in the 2006–2009 Behavioral Risk Factor Surveillance Survey

**DOI:** 10.1186/1756-0500-5-253

**Published:** 2012-05-23

**Authors:** Alberto J Caban-Martinez, Evelyn P Davila, Byron L Lam, Kristopher L Arheart, Kathryn E McCollister, Cristina A Fernandez, Manuel A Ocasio, David J Lee

**Affiliations:** 1 Department of Epidemiology & Public Health, University of Miami, Miller School of Medicine, Clinical Research Building, Room 1075, 1120 N.W. 14th Street, 10th Floor (R-669), Miami, FL, 33136, USA; 2 Department of Ophthalmology, Miller School of Medicine, University of Miami, Miami, FL, USA

**Keywords:** Vision Health, Eye Care, Older Adults, Epidemiology, National Survey

## Abstract

**Background:**

Research has suggested that adults 40 years old and over are not following eye care visit recommendations. In the United States, the proportion of older adults is expected to increase drastically in the coming years. This has important implications for population ocular disease burden, given the relationship between older age and the development of many ocular diseases and conditions. Understanding individual level determinants of vision health could support the development of tailored vision health campaigns and interventions among our growing older population. Thus, we assessed correlates of eye care visits among participants of the Behavior Risk Factor Surveillance System (BRFSS) survey. We pooled and analyzed 2006–2009 BRFSS data from 16 States (N = 118,075). We assessed for the proportion of survey respondents 40 years of age and older reporting having visited an eye care provider within the past two years, two or more years ago, or never by socio-demographic characteristics.

**Results:**

Nearly 80% of respondents reported an eye care visit within the previous two years. Using the ‘never visits’ as the referent category, the groups with greater odds of having an ocular visit within the past two years included those: greater than 70 years of age (OR = 6.8 [95% confidence interval = 3.7–12.6]), with college degree (5.2[3.0–8.8]), reporting an eye disease, (4.74[1.1–21.2]), diagnosed with diabetes (3.5[1.7–7.5]), of female gender (2.9[2.1–3.9]), with general health insurance (2.7[1.8–3.9]), with eye provider insurance coverage (2.1[1.5–3.0]), with high blood pressure (1.5[1.1–2.2]), and with moderate to extreme near vision difficulties (1.42[1.11–2.08]).

**Conclusion:**

We found significant variation by socio-demographic characteristics and some variation in state-level estimates in this study. The present findings suggest that there remains compliance gaps of screening guidelines among select socio-demographic sub-groups, as well as provide evidence and support to the CDC’s Vision Health Initiative. This data further suggests that there remains a need for ocular educational campaigns in select socio-demographic subgroups and possibly policy changes to enhance insurance coverage.

## Background

The proportion of persons older than 40 years old in the United States is expected to increase drastically in the coming years [1]. This has important implications for population ocular disease burden, given the relationship between older age and the development of many ocular diseases and conditions [2]. Among older adults, impaired visual acuity is generally associated with negative health outcomes, such as decreased functional capacity and quality of life, sometimes impinging on the ability to live an autonomous life [3-6]. Impaired visual acuity has also been associated with an inability to perform both basic and instrumental activities of daily living, including work, driving safely or obtaining a driver’s license, and even an increase risk of falls and other accidental injuries [7-11]. Older adults may also be unaware of impaired vision, given the relatively insidious development of vision change symptoms over time. Therefore, periodic screening eye exams are paramount to identifying vision changes, preventing the progression of visual disorders and rendering appropriate vision care that improves quality of life and functional capacity in our adult populations [12].

The American Academy of Ophthalmology (AAO) and the American Optometric Association (AOA) recommend comprehensive eye examinations by ophthalmologists or optometrists for adults with no signs or risk factors to be conducted as a baseline at the age 40 years [13-15]. Current recommendations by the AOA suggest that 40 to 54 year olds without risk factors should be examined every two to four years, those age 55 to 64 every one to three years, and those over 65 every one to two years. In addition, they recommend that any patient at higher risk for developing disease, based on ocular, medical, or family history should have periodic examinations determined by their individual risk and eye care provider. Understanding the distribution of eye and vision conditions, as well as the sociodemographic correlates of adults who visit eye care providers, may inform eye care efforts and allow for tailored eye care intervention efforts. Therefore we: 1) Examine sociodemographic correlates of eye care provider visits among adults 40 and older; 2) Describe the strength of the association between sociodemographic characteristics associated with eye care provider visit; and 3) Provide state-level prevalence estimates of eye care provider visits.

## Methods and Materials

### Study Population and Data Source

The Behavioral Risk Factor Surveillance System (BRFSS), developed by the Centers for Disease Control and Prevention (CDC), is an ongoing, state-based, random-digit-dialed telephone survey that collects information on health risk behaviors, preventive health practices, and health care access primarily related to chronic disease and injury among a non-institutionalized, civilian adult population aged 18 years and older [16]. This state-administered survey is comprised of an annual set of core health-related questions asked in all 50 states, the District of Columbia, Guam, Puerto Rico, and the Virgin Islands States, as well as a set of optional modules with specific health-related questions that are fielded by the state, based on priorities and financial resources.

Beginning in 2006, the optional Visual Impairment and Access to Eye Care module was included in the survey to assess prevalence of self-reported visual impairment, eye disease, eye injury, and lack of eye care insurance and eye examination among persons aged 40 years and older. Details about its purpose, sampling methods, data collection, and reporting are available elsewhere [16].

We pooled data from the 2006–2009 BRFSS for all adults aged 40 years and older participating in the Visual Impairment and Access to Eye Care module administered by the following States: Alabama, Alaska, Connecticut, Florida, Georgia, Indiana, Iowa, Missouri, New Mexico, New York, North Carolina, Ohio, Tennessee, Texas, West Virginia and Wyoming (N = 118,075). The median survey cooperation rate from 2006–2009 ranged from 72.1% to 75.0% [16].

### Measures

#### Dependent variable: Eye Care Provider Visit

Participants were asked “When was the last time you had your eyes examined by any doctor or eye care provider?” and responded either within the past month, within the past year, within the past 2 years, 2 or more years ago, or never. We grouped respondents into three outcomes 1) having visited an eye care provider within the past two years, 2) two or more years ago, or 3) never.

#### Independent variables

Age was categorized into four age groups: 40–49, 50–59, 60–69, and > 70 years old. Additional sociodemographic covariates included: gender, race/ethnicity (grouped as white non-Hispanic, black non-Hispanic, other non-Hispanic, and Hispanic), highest educational attainment (categorized as those reporting less than a high school diploma, a high school diploma, attending some college, or having graduated from college), marital status (categorized as being married/living with a partner, widowed/divorced/separated, or being single).

Participants were also classified based on general health insurance coverage (yes or no), and eye care provider insurance coverage (yes or no). Body mass index (BMI) was based on reported height and weight and was categorized as: (1) neither overweight nor obese [< 25 kg/m2]; (2) overweight [25–29.9 kg/m2]; or (3) obese [> = 30 kg/m2]. Participants were also classified as diabetic or hypertensive if they indicated they were told by a health care professional that they had these conditions.

Survey participants reported on far vision difficulties (categorized as dichotomous “little to none” or “moderate to extreme”), near vision difficulties (also categorized as a dichotomous “little to none” versus “moderate to extreme”), and their eye disease status based on the respondents affirmation to either having been told by a doctor they had cataracts, glaucoma, or age-related macular degeneration (categorized into yes or no if the respondent indicated they had any of the listed ocular diseases).

### Statistical Analyses

We analyzed data from individuals who had visited an eye care provider within the past two years, two or more years ago, or never. Prevalence estimates for eye care provider visits and the proportion of participants categorized by frequency of eye care visits across a number of socio-demographic and health-related characteristics are presented in Table 1. Specific state-level prevalence estimates of eye care provider visits are presented in Table 2. A polychotomous logistic regression model with adjustment for survey design tested the association between categories of eye care provider visit frequency and age groups controlling for select socio-demographic characteristics (Table 3).

**Table 1:** Socio-Demographic Characteristics and Frequency of Reported Eye Care Provider Visit of Adults (≥ age 40)- The 2006–2009 Behavioral Risk Factor Surveillance Survey** (N = 118,075)†

	**WITHIN THE PAST TWO YEARS**			**2 OR MORE YEARS AGO**			**NEVER**		
**Demographics**	**Sample N**	**Estimated AnnualPopulation**	**Percent ± Standard Error**	**Sample N**	**Estimated Annual Population**	**Percent ± Standard Error**	**Sample N**	**Estimated AnnualPopulation**	**Percent ± Standard Error**
**Total**	95,007	42,657,170	79.5 ± 0.2	21,483	9,942,174	18.8 ± 0.2	1,585	950,723	1.8 ± 0.1
**Age**									
40-49 years old	18,899	12,295,708	29.2 ± 0.3	6,478	4,183,454	42.1 ± 0.7	823	602,806	63.4 ± 2.2
50-59 years old	24,971	11,798,975	28.0 ± 0.3	6,693	2,969,638	29.9 ± 0.6	483	257,826	27.1 ± 2.1
60-69 years old	23,189	8,419,706	20.0 ± 0.2	4,809	1,692,169	17.0 ± 0.5	181	62,891	6.6 ± 0.8
> 70 years old	26,888	9,615,336	22.8 ± 0.2	3,306	109,691	11.0 ± 0.4	86	27,200	2.9 ± 0.7
**Gender**									
Male	33,350	19,293,135	45.2 ± 0.3	9051	5,281,995	52.6 ± 0.7	866	586,433	61.1 ± 2.3
Female	61,657	23,364,035	54.8 ± 0.3	12,432	4,761,838	47.4 ± 0.7	719	372,763	38.9 ± 2.3
**Race/Ethnicity**									
White, Non-Hispanic	76,740	32,311,744	76.5 ± 0.3	16,923	7,483,135	75.2 ± 0.6	1,019	508,326	53.9 ± 2.4
Black, Non-Hispanic	9,951	4,178,925	9.9 ± 0.2	2,362	986,422	9.9 ± 0.4	207	92,745	9.8 ± 1.1
Other, Non-Hispanic	2,936	1,863,621	4.4 ± 0.1	771	511,119	5.1 ± 0.4	85	59,147	6.3 ± 1.0
Hispanic	4,389	3,864,274	9.2 ± 0.3	1,210	966,198	9.7 ± 0.5	250	283,682	30.1 ± 2.5
**Marital Status**									
Married/Living with Partner	54,074	29,522,056	69.4 ± 0.3	11,413	6,656,542	66.5 ± 0.6	904	690,728	72.2 ± 1.8
Widowed, Divorced or Separated	34,502	10,699,810	25.2 ± 0.3	8,046	2,521,895	25.2 ± 0.5	502	183,293	19.2 ± 1.5
Single	6,054	2,294,113	5.4 ± 0.1	1,948	833,994	8.3 ± 0.3	175	82,020	8.6 ± 1.1
**Education**									
Less than High School	10,220	4,249,803	10.0 ± 0.2	3291	1,461,052	14.6 ± 0.5	439	302,500	31.6 ± 2.4
High School Diploma	30,293	12,127,596	28.5 ± 0.3	7632	3,265,457	32.6 ± 0.6	610	309,840	32.3 ± 2.0
Attended College	24,703	11,109,821	26.1 ± 0.3	5329	2,429,357	24.2 ± 0.6	271	162,584	17.0 ± 1.9
Graduated from College	29,570	15,051,606	35.4 ± 0.3	5182	2,865,158	28.6 ± 0.7	262	182,904	19.1 ± 1.9
**Body Mass Index**									
Normal Weight	30,432	13,540,061	33.1 ± 0.3	6754	3,129,859	32.4 ± 0.7	523	310,752	35.8 ± 2.4
Overweight	34,287	15,936,469	38.9 ± 0.3	7624	3,650,659	37.8 ± 0.7	527	312,445	36.0 ± 2.3
Obese	26,140	15,936,469	28.0 ± 0.3	6213	2,881,679	29.8 ± 0.7	432	245,332	28.2 ± 2.2
**Eye Insurance Status**									
Has Eye Insurance	54330	25,760,864	61.5 ± 0.3	7949	4,173,169	43.5 ± 0.7	474	284,942	31.4 ± 2.2
No Eye Insurance	38931	16,118,968	38.5 ± 0.3	12541	5,429,954	56.5 ± 0.7	1,006	622,259	68.6 ± 2.2
**Health Insurance Status**									
Has Health Insurance	88290	39,164,162	92.0 ± 0.2	17408	7,948,234	79.3 ± 0.6	1,015	542,122	56.8 ± 2.4
No Health Insurance	6558	3,392,391	8.0 ± 0.2	4019	2,077,566	20.7 ± 0.6	561	412,791	43.2 ± 2.4
**Far Vision Difficulties**									
Has Little to None	88,804	40,142,531	95.4 ± 0.1	19767	9,357,790	94.5 ± 0.3	1,491	910,441	96.7 ± 0.8
Has Moderate to Extreme	4,906	1,948,998	4.6 ± 0.1	1392	546,716	5.5 ± 0.3	64	31,301	3.3 ± 0.8
**Near Vision Difficulties**									
Has Little to None	82,508	39,234,257	88.4 ±0.2	17049	8,081,003	82.1 ± 0.5	1,188	746,434	80.3 ± 1.7
Has Moderate to Extreme	11,092	4,866,227	11.6 ± 0.2	3973	1,765,173	17.9 ± 0.5	351	183,319	19.7 ± 1.7
Eye Disease Status*									
Has Cataract	17,228	6,227,833	16.5 ± 0.2	1372	437,948	4.6 ± 0.2	15	2,961	0.3 ± 0.1‡
Has Glaucoma	5,947	2,368,372	5.6 ± 0.1	343	138,714	1.4 ± 0.1	4	3,750	0.4 ± 0.2‡
Has Age-Related Macular Degeneration	5,242	2,093,948	5.0 ± 0.1	454	212,879	2.2 ± 0.2	7	4,056	0.4 ± 0.3‡
Chronic Disease Status*									
Has Diabetes	15,175	6,141,139	14.7 ± 0.2	2053	781,008	7.9 ± 0.3	76	36,519	3.9 ± 0.8
Has Hypertension	15,665	3,818,886	43.8 ± 0.5	3014	764,411	40.0 ± 0.9	155	47,163	26.6 ± 3.1

† = Differences in sub-total population sample may not add to total due to item non-response or missing.

‡ = Estimate does not meet National Center for Health Statistic’s standard of reliability or precision given the relative standard error was greater than 30%.

* = Sample n refers to the number of persons with the condition; Presented estimates are row percents of the total sample for condition (i.e. cataract, diabetes).

** = States surveyed = Alabama, Alaska, Connecticut, Florida, Georgia, Indiana, Iowa, Missouri, New Mexico, New York, North Carolina, Ohio, Tennessee, Texas, West Virginia, and Wyoming.

**Table 2:** State Level Estimates of Frequency of Reporting Eye Care Provider Visit for adults (≥ age 40 years)- The 2006–2009 Behavioral Risk Factor Surveillance Survey (N = 118,075)

	**WITHIN THE PAST TWO YEARS**			**2 OR MORE YEARS AGO**			**NEVER**		
**State Specifics**	**Sample N**	**Estimated AnnualPopulation**	**Percent ± Standard Error**	**Sample N**	**Estimated AnnualPopulation**	**Percent ± Standard Error**	**Sample N**	**Estimated AnnualPopulation**	**Percent ± Standard Error**
Alabama	12,484	1,654,872	79.0 ± 0.5	2,870	401,267	19.2 ± 0.4	224	38,015	1.8 ± 0.2
Alaska	2,835	1,947,321	81.6 ± 1.2	628	415,073	17.4 ± 1.2	41‡	25,254	1.1 ±0.4‡
Connecticut	4,026	1,380,228	82.0 ± 0.8	754	292,665	17.4 ± 0.7	35‡	10,970	0.7 ± 0.1‡
Florida	6,624	6,954,837	81.6 ± 0.6	1,310	1,369,507	16.1 ± 0.6	138	193,798	2.3 ± 0.3
Georgia	8,805	2,858,914	78.1 ± 0.5	2,072	741,347	20.2 ± 0.5	153	61,631	1.7 ± 0.2
Indiana	8,350	2,118,487	78.2 ± 0.6	2,102	554,609	20.5 ± 0.5	108	37,246	1.4 ± 0.2
Iowa	7,060	1,092,764	80.8 ± 0.5	1,445	240,577	17.8 ± 0.5	91	19,632	1.5 ± 0.2
Missouri	2,966	1,908,869	73.4 ± 1.0	935	638,409	24.6 ± 0.9	60	52,018	2.0 ± 0.3
New Mexico	3,651	640,405	75.5 ± 0.8	981	185,502	21.9 ± 0.8	96	22,269	2.6 ± 0.3
New York	7,566	6,864,984	82.0 ± 0.5	1,475	1,379,011	16.5 ± 0.5	97	124,990	1.5 ± 0.2
North Carolina	9,876	3,160,247	78.2 ± 0.5	2,262	798,792	19.8 ± 0.5	178	82,470	2.0 ± 0.2
Ohio	3,383	1,996,997	78.8 ± 1.2	787	515,389	20.3 ± 1.1	38‡	23,356	0.9 ± 0.3‡
Tennessee	5,928	2,231,474	80.5 ± 0.7	1,142	477,692	17.2 ± 0.7	129	61,190	2.2 ± 0.3
Texas	3,777	6,950,523	77.7 ± 1.0	893	1,800,936	20.1 ± 1.0	97	189,467	2.1 ± 0.3
West Virginia	2,688	707,781	78.0 ± 0.8	673	185,309	20.4 ± 0.8	45	14,234	1.6 ± 0.2
Wyoming	4,988	188,459	78.9 ± 0.6	1,154	47,743	20.0 ± 0.6	55	2,649	1.1 ± 0.2

‡ = Estimate does not meet National Center for Health Statistic’s standard of reliability or precision given the relative standard error was greater than 30%.

**Table 3:** Polytomous logistic regression for Predictors of Visiting Eye Care Provider for adults (≥ age 40 years)- The 2006–2009 Behavioral Risk Factor Surveillance Survey* (N = 118,075)

**PREDICTORS**	OCULAR VISIT WITHIN THE PAST TWO YEARS**		OCULAR VISIT ≥ TWO YEARS**	
	OR	95% CI	OR	95% CI
**Age***(ref = 40–49 years old)*	1.00		1.00	
50-59 years old	**2.18**	**1.55-3.09**	**1.57**	**1.11-2.22**
60-69 years old	**4.65**	**2.25-7.85**	**3.05**	**1.79-5.17**
≥ 70 years old	**6.82**	**3.70-12.57**	**3.08**	**1.65-5.76**
**Gender***(ref = male)*	1.00		1.00	
Female	**2.87**	**2.12-3.91**	**2.22**	**1.62-3.03**
**Race/Ethnicity***(ref = White, Non-Hispanic)*	1.00		1.00	
Black, Non-Hispanic	0.74	0.73-1.29	0.64	0.37-1.12
Other, Non-Hispanic	0.90	0.42-1.93	0.72	0.33-1.57
Hispanic	0.91	0.41-2.01	0.54	0.23-1.27
**Marital Status***(ref = Married, Living with Partner)*	1.00		1.00	
Widowed, Divorced or Separated	1.07	0.74-1.57	1.43	0.97-2.09
Single	1.43	0.98-2.09	1.77	0.92-3.42
**Education***(ref = Less than High School)*	1.00		1.00	
High School Diploma	**1.89**	**1.20-2.97**	1.25	0.79-1.97
Attended College	**3.03**	**1.81-5.08**	**1.85**	**1.10-3.11**
Graduated from College	**5.16**	**3.02-8.81**	**2.35**	**1.37-4.04**
**Eye Provider Insurance***(ref = No Eye Insurance)*	1.00		1.00	
Yes	**2.08**	**1.46-2.95**	1.05	0.74-1.50
**General Health Insurance***(ref = No Health Insurance)*	1.00		1.00	
Yes	**2.66**	**1.80-3.93**	**1.76**	**1.19-2.61**
**Diabetes Status***(ref = no diabetes)*	1.00		1.00	
Yes, has diabetes	**3.53**	**1.66-7.50**	**2.24**	**1.06-4.80**
**High Blood Pressure Status***(ref = no high blood pressure)*	1.00		1.00	
Yes, has high blood pressure	**1.54**	**1.08-2.20**	**1.63**	**1.14-2.34**
**Far Vision Difficulties***(ref = has little to moderate)*	1.00		1.00	
Has Moderate to Extreme	1.68	0.74-3.82	1.58	0.69-3.62
**Near Vision Difficulties*(****ref = has little to moderate)*	1.00		1.00	
Has Moderate to Extreme	**1.42**	**1.11-2.08**	0.92	0.61-1.40
**Eye Disease Status*(****ref = has no Eye Disease)*	1.00		1.00	
Has eye disease (e.g. cataract, glaucoma, and/or ARMD)	**4.74**	**1.06-21.22**	1.80	0.40-8.11

** Reference group = Never.

*Statistically significant findings in bold.

All analyses were performed using SPSS 17 Complex Samples for Survey Analysis (SPSS Inc., Chicago, IL, 2008) to account for multiple stages of sampling, stratification, and clustering. An alpha level of 0.05 was considered statistically significant. All analyses in this study were weighted according to the standard procedures for analyzing sample survey data [16]. For pooled prevalence estimates, sample weights were adjusted to account for the aggregation of data over multiple survey years for each state by dividing the original weight by the number of years a state was included in the study period (i.e. Alabama, Georgia, Indiana, Iowa, Missouri, New York, North Carolina, Ohio, Tennessee) [16]. The study protocol was approved by the University of Miami’s Institutional Review Board.

## Results

The study population represented an estimated 53.6 million U.S. adults annually from 2006–2009. The prevalence of visiting an eye care provider within the past two years, two or more years ago, or never by sociodemographic characteristics, are presented in Table 1. Overall, 79% (representing an estimated annual 42.6 million U.S. adults 40 years of age of older among all 16 States surveyed) reported visiting an eye care provider within the previous two years, 19% reported two or more years ago, and 2% reported that they had never seen an eye care provider. Over 40% of those who reported visiting an eye care provider two or more years ago were 40 to 49 years old. Over 63% of those who reported never having been to an eye provider fell into this age category, indicating that adults in this age range were less likely to be compliant with screening recommendations. State-level estimates varied slightly across eye care provider visit interval category (Table 2). Respondents residing in Connecticut (82.0% ± 0.8) and New York (82.0% ± 0.5) had the highest prevalence for visiting an eye care provider within the past two years, while respondents from Missouri (73.4% ± 1.0) and New Mexico (75.5% ± 0.8) had the lowest.

### Eye care visit within the past two years

In the polytomous multiple logistic regression (See Table 3), using the never visits as the referent category, the strongest associations with report of an ocular visit within the past two years was being greater than 70 years of age relative to 40–49 years (OR = 6.8 [95% confidence interval = 3.7–12.6]), having graduated from college relative to those who did not complete high school (5.2 [3.0–8.8]), having an eye condition (e.g. cataract, glaucoma, and/or age-related macular degeneration) relative to no eye condition (4.74 [1.1–21.2]), having diabetes (3.5 [1.7–7.5]), being female (2.9 [2.1–3.9]), having general health insurance (2.7 [1.8–3.9]), having the availability of eye provider health insurance coverage (2.1 [1.5–3.0]), having high blood pressure (1.5 [1.1–2.2]), and having moderate to extreme near vision difficulties relative to little to moderate difficulties (1.42 [1.11–2.08]).

### Eye care visit greater than two years ago

Using the never visits as the referent category, the strongest associations with report of an ocular visit greater than two years ago was being greater than 70 years of age relative to 40–49 years (OR = 3.1 [1.7–5.8]), having graduated from college relative to those who did not complete high school (2.4 [1.4–4.0]), having diabetes (2.2 [1.1–4.8]), being female (2.2 [1.6–3.0]), having general health insurance (1.8 [1.2–2.6]), and having high blood pressure (1.6 [1.1–2.3]).

## Discussion

We found significant variation by socio-demographic characteristics and some variation in state-level estimates. Correlates of visiting an eye care provider included: being older (e.g. 70 years of age or older), being female, having higher educational attainment, having general health and eye care insurance, being diagnosed with diabetes or high blood pressure, reporting an eye condition such as cataract, glaucoma, or age-related macular degeneration, and having near vision difficulties. To our knowledge, no previous studies have used multistate-level data to test the association between sociodemographic characteristics and report of eye care provider visit. Our findings are consistent with studies from the Los Angeles Latino Eye Study, which reported that select socio-demographic characteristics are strongly associated with more frequent eye care provider visits among Latinos, for example: age, educational attainment, general health and eye insurance status, and co-morbidities [17].

A visit to an eye care provider within the past two years in this study was associated with a number of variables in our multivariable analysis. Among predisposing variables, older age, female gender, and more education were independently associated with greater use of provider eye care service. These results are consistent with previous research showing that women and older individuals are more likely to use vision health services than their male and younger counterparts [18]. Previous literature has also shown that education is associated with greater use of eye care [18]. Nonetheless, these relatively strong independent variables for eye care, such as the social predisposing variable (education) and the enabling variable (insurance status), suggested that the least educated and uninsured were also the least likely to use eye care services. These groups deserve focused attention in any interventions designed to increase eye care utilization rates in these socio-demographic subgroups.

Other factors correlated with greater odds of visiting an eye care provider within the past two years included: having primary ocular disease such as cataract, glaucoma, or age-related macular degeneration, (although the 95% confidence interval for this estimate was large rates (1.06–22.22)). These findings are also similar to results from the Blue Mountains Eye Study in Australia, which included clinical eye examinations [19]; they reported that blue mountain participants with a history of diabetes, hypertension or with any major eye pathology, including moderate to severe myopia, were significantly more likely to have seen an ophthalmologist in the past 2 years. We found that general health and eye insurance were important enabling variables, therefore, we conducted a stepwise regression analyses to identify indicators of eye care for the subgroup of participants with general health and vision insurance. Significant indicators of eye care in the past 24 months (*P* < 0.05) were: (1) Having a larger number of chronic conditions, (2) Having near vision difficulties, (3) Having a higher level of education, (4) Being of female gender, and (5) Being of older age.

We found some variation in state-level estimates of eye care provider visits. Among respondents attending an eye care provider visit within the past two years, adults from Connecticut and New York had the highest estimates for visiting an eye care provider, while respondents from Missouri and New Mexico had the lowest. Studies suggest that state variation in health care visits is driven by underlying economic and demographic factors, such as the employment makeup in the state (e.g., firm size, industry and occupation, and the degree of unionization), eligibility requirements for public programs such as Medicaid, and the demographic/socioeconomic composition of state residents [20-22]. State variation in employer-sponsored coverage appears to be driven, in part, by employee characteristics, such as industry and length of time spent with an employer, and local labor market characteristics, such as state-level unionization [23]. Given that general health and eye care insurance were associated with report of recent eye care visits, all findings consistent were with those reported by Zhang et al. [24], and variations in economic and labor mixes in the each state could be driving the observed differences.

### Strengths and Limitations

This study adds to the literature by being the first to describe the association between eye care provider visits and socio-demographic characteristics using recent population-based data across multiple US states. We were also able to identify the contributions of several important variables (e.g., health and eye insurance status) to these relationships. Although the BRFSS data have been found to provide valid and reliable estimates as compared with the national household surveys [25], our study has several limitations. First, the cross-sectional design does not allow for causal inferences. Since BRFSS is a telephone based survey, there is the possibility of non-response bias. In addition, the survey used for this study was based on self-reported data and data on the type and quality of health care visits were not available. Studies have shown that self-reported data, particularly of less socially desirable behaviors, are subject to limitations of underreporting and recall bias.

## Conclusion

Published recommendations by professional organizations for screening and comprehensive eye examinations by ophthalmologists and optometrist have existed for many years [13-15]. However, the present findings suggest that there remain compliance gaps for these screening guidelines among select socio-demographic sub-groups, as well as provide evidence and support to the CDC’s Vision Health Initiative [26]. Impaired vision in aging adults may not be recognized or may remain unreported because vision changes can be relatively subtle, progress slowly over time, or occur in persons with cognitive dysfunction or other co-morbidities. However, even mildly impaired visual acuity can be associated with decreased quality of life and functional capacity and increase the likelihood of accidents and related injuries [3-6]. Vision screening interventions and services targeted at at-risk subgroups such as the uninsured are needed to address population vision health.

## Competing interests

The authors declare that they have no competing interests

## Authors’ contributions

AJCM: conceived the study, participated in its design, coordination, performed statistical analyses, and co-drafted the manuscript. EPD: participated in the design of the study, performed statistical analysis and helped with the manuscript draft. BLL: participated in the design of the study, performed statistical analysis and helped with the manuscript draft. KLA: participated in the design of the study, performed statistical analysis and helped with the manuscript draft. KEM: participated in analysis of the study results and helped with the manuscript draft CAF: participated in analysis of the study results and helped with the manuscript draft. MAO: participated in analysis of the study results and helped with the manuscript draft DJL: conceived the study, participated in its design, coordination, performed statistical analyses, and co-drafted the manuscript. All authors read and approved the final manuscript.

## Funding

Support for this study was provided by National Eye Institute grant (R21-EY019096).
